# How bureaucracy is bleeding science dry: international observational research under the General Data Protection Regulation

**DOI:** 10.1016/j.lanepe.2024.101200

**Published:** 2024-12-26

**Authors:** Derek P. de Winter, Nina A.M. Houben, Enrico Lopriore

**Affiliations:** a Division of Neonatology, Willem-Alexander Children's Hospital, Leiden University Medical Center, Leiden, the Netherlands; b Sanquin Research, Sanquin Blood Supply Foundation, Amsterdam, the Netherlands

International research collaborations play an essential role in medical science. Many clinical research questions can be answered by using routinely collected patient data in observational studies, specifically studies assessing implementation of existing evidence, variations in current practice, and diseases or interventions with a low prevalence. Despite the potential high-gain, these low-risk observational studies have been increasingly hampered by administrative burdens and varying interpretations of the General Data Protection Regulation (GDPR). In recent years, we initiated two large international observational studies that have given us first-hand experience in the practical challenges of conducting research under the GDPR.^1^, ^2^, ^3^

The GDPR, effective in European Union (EU) Member States since 2018, regulates the processing of personal data and the free movement of such data.^4^ The principles underlying this and other regulations, such as ensuring patient safety, protecting privacy, and safeguarding research quality and integrity, are of paramount importance. Yet, its interpretation varies widely across European countries. Not only between countries, but also between centers within the same country, resulting in a multitude of ethics applications. This complexity jeopardizes the feasibility of many observational studies, undermining their potential as a pragmatic approach to identify opportunities to improve patient care.

In practice, this often leaves researchers drowning in paperwork, and results in frustration (at least in the three of us), high costs and inefficiency. More importantly, it discourages young investigators from initiating large-scale international studies, as they are commonly bound to a limited research contract. This often leads researchers to limit the number of centers involved to reduce the workload. However, the quality and generalizability of study results are greatly enhanced by a representative sample of participating centers. Effective collaborations should include a diverse range of centers beyond those in countries with higher research budgets, more standardized procedures or more dedicated research staff. Additionally, these obstacles are reinforced by budget cuts in many health care institutions that were forced to dismiss support staff crucial in facilitating such studies. Although well-intentioned, many of these rules make it nearly impossible to conduct large-scale research, and demotivate researchers.

In our experience resulting from two large international studies, the different interpretations of the GDPR resulted in some centers requiring informed consent, others using an opt-out approach, or waiving the need of consent, even though all were participating in an equally low-risk study. Similarly we observed that requirements for patient information leaflets varied across centers, which resulted in inconsistent information being provided to parents within the same study. Performing observational research under the GDPR feels like trying to cross a busy street blinded by paperwork, with more forms handed to you at every step, and each crossing guard enforcing different rules. One says, ‘Stop for a green light,’ another says, ‘Go on orange,’ and a third insists you ‘Wait for blue’, even though such a light does not exist. All the while, you cannot shake the feeling that taking a nearby crosswalk would simplify everything. Thanks to a dedicated full-time PhD position, we were able to navigate this busy street and bring these studies to life, identifying concrete opportunities to directly improve patient care. However, such positions remain a rare privilege in many cases. And even with a dedicated research contract, it has sometimes driven us deep into despair.

In conclusion, we feel that these excessive administrative demands often fail to enhance quality, but instead have the opposite effect by consuming time, resources and energy unnecessarily. Addressing these challenges requires more than local solutions, but demands both national and international standardization in interpreting and applying regulations. However, merging and unifying ethical approval for low-risk observational research within the same country could already significantly reduce the number of applications. Additionally, it requires changes in regulatory frameworks to align better with practical realities, by applying risk differentiation to ensure that regulations are proportionate to the level of risk associated. Without any change, the potential of observational international collaborations will bleed dry.

## Contributors

DW and NH contributed equally to this correspondence. DW and NH have conceptualized the correspondence, EL supervised the project. DW and NH wrote the first draft of the correspondence. All authors critically reviewed the draft and approved the final version prior to submission. Data curation, formal analysis, funding acquisition, investigation, methodology, project administration, resources, software were not applicable for this correspondence.

## Declaration of interests

The authors declare no conflicts of interest.
